# Transcriptomic Time-Course Sequencing: Insights into the Cell Wall Macromolecule-Mediated Fruit Dehiscence during Ripening in *Camellia oleifera*

**DOI:** 10.3390/plants12183314

**Published:** 2023-09-20

**Authors:** Yu Sheng, Xiaohua Yao, Linxiu Liu, Chunlian Yu, Kunxi Wang, Kailiang Wang, Jun Chang, Juanjuan Chen, Yongqing Cao

**Affiliations:** 1 State Key Laboratory of Tree Genetics and Breeding, Key Laboratory of Tree Breeding of Zhejiang Province, Research Institute of Subtropical Forestry, Chinese Academy of Forestry, Hangzhou 311400, China; shengyu2000@foxmail.com (Y.S.); lxliu9968@163.com (L.L.);; 2 Quzhou Doctoral Innovation Workstation, Changshan Country Oil Tea Industry Development Center, Quzhou 323900, China; yuchunlian0116@126.com (C.Y.); kunxiwang2023@163.com (K.W.); 3 Faculty of Forestry, Nanjing Forestry University, Nanjing 210037, China

**Keywords:** *Camellia oleifera*, transcriptome, cell wall, fruit dehiscence

## Abstract

*Camellia oleifera* (*C. oleifera*), one of the world’s four major edible woody oil crops, has been widely planted in southern China’s subtropical region for the extremely high nutritional and health benefits of its seed oil. Timing and synchronization of fruit dehiscence are critical factors influencing the oil output and quality, as well as the efficiency and cost of harvesting *C. oleifera*, yet they extremely lack attention. To gain an understanding of the molecular basis underlying the dehiscence of *C. oleifera* fruit, we sampled pericarp–replum tissues containing dehiscence zones from fruits at different developmental stages and performed time-series transcriptomic sequencing and analysis for the first time. Statistical and GO enrichment analysis of differentially expressed genes revealed that drastic transcriptional changes occurred over the last short sampling interval (4 days, 18th–22nd October), which directed functional classifications link to cell wall and cell wall macromolecule activity. WGCNA further showed that factors controlling cell wall modification, including endo-1,3;1,4-beta-D-glucanase, WAT1-like protein 37, LRR receptor-like serine/threonine-protein kinase, and cellulose synthase A catalytic subunit, were identified as core members of the co-expression network of the last stage highly related modules. Furthermore, in these modules, we also noted genes that were annotated as coding for polygalacturonase and pectinesterase, two pectinases that were expected to be major players in cell separation during dehiscence. qRT-PCR further confirmed the expression profiles of these cell wall modification relating factors, which possessed a special high transcriptional abundance at the final stage. These results suggested the cell wall associated cell separation, one of the essential processes downstream of fruit dehiscence, happened in dehiscing fruit of *C. oleifera* during ripening. Hydrolases acting on cell wall components are good candidates for signal mediating dehiscence of *C. oleifera* fruit. In conclusion, our analysis provided insights into the cell wall macromolecule-mediated fruit dehiscence during ripening in *C. oleifera*.

## 1. Introduction

Fruit dehiscence is a critical process for seed dispersal affecting the completion of the propagation cycle of many plant species, and is tightly associated with yield losses in agriculture. Preharvest fruit dehiscence causes seeds to be shed before or during harvest, bringing substantial yield losses [1,2]. Conversely, delayed dehiscence is not conducive to harvesting, and the accumulation of dry matter or desired components will be reduced or changed due to the respiration of seeds [2]. The dehiscing process of fruit has been well studied in plants of legumes and crucifers, such as soybean, *Arabidopsis thaliana* (*A. thaliana*), and *Brassica napus* (*B. napus*) [1,3]. The legume pod has a single seed-attaching carpel and lacks a replum [4]. By contrast, the fruit of rapeseed and *Arabidopsis* share multiple similarities in the shattering process, as well as a similar silique structure [2,5], which consists of two fused valves (carpels), connected with the replum [4]. 

Environmental factors, such as temperature and humidity, play as essential triggers in fruit dehiscence and affect the yield loss of major crops such as canola and soybean. Structural factors are crucial for the occurence of dehiscence [6]. The dehiscence zone (DZ), a common and important structure, occurs along the edge between the valves, or the valve and the replum, and mediates the indehiscence or dehiscence of matured fruit. An abnormal phenotype of fruit dehiscence is usually accompanied by special tissue morphology of the DZ, including developmental defects [2,6]. The cell separation process in the DZ, enabling fruit dehiscence, is coordinately controlled by multiple regulators. In *Arabidopsis*, the DZ develops from the lignified and non-lignified cells located on the valve margin [7]. In the current basic framework, defined by six transcription factors of the genetic network controlling dehiscence of *A. thaliana* fruit [8], *INDEHISCENCE* (*IND*) and *ALCATRAZ* (*ALC*) function in valve margin identity. The former directs the development of the lignified and the separation layer, and the latter specifies the development of the separation layer [2,9]. *SHATTERPROOF1* (*SHP1*) and *SHP2*, related to lignin deposition, redundantly and positively regulate *IND* and *ALC* [2,8]. *FRUITFULL* (*FUL*) and *REPLUMLESS* (*RPL*) are, respectively, activated in the valves and the replum, to restrict and negatively regulate the expression of the above-mentioned DZ genes at the valve margin [2,8]. During the ripening process, adhesion resulting from pectin-rich deposits among cells in the separation layer is weakened by hydrolases secreted by thin-walled cells [10,11]. Previous studies have characterized that the two pectinases, ARABIDOPSIS DEHISCENCE ZONE POLYGALACTURONASE1 (ADPG1) and ADPG2, are essential for fruit dehiscence in *A. thaliana* [12], while the cellulase gene *CELLULASE6* (*CEL6*) and the hemicellulase gene *MANNANASE7* (*MAN7*) affect cell wall degradation in the separation layer of the DZ to promote the silique dehiscence [13]. Once the mechanical tensions driven by the differential shrinkage between the lignified and non-lignified tissues within the fruit exceed the cell adhesion in the separation layer, this allows the valve to be easily separated off the replum at maturity [2]. Currently, increasing factors related to the development of leaf, flower, lateral organ, meristem, and plant hormones (ethylene, auxin, cytokinins, and gibberellin) have been continuously updated to the regulatory networks of silique dehiscence [2].

*Camellia oleifera* (*C. oleifera*), an evergreen edible woody oil species belonging to the genus *Camellia* (Theaceae), is widely cultivated for oil production in subtropical montane regions in China. It is estimated that the planting area of *C. oleifera* will reach 6 million hectares in 2025, providing a gross production value of CNY 400 billion (China Green Times, 2023). Seeds, as the carrier of the excellent nutritional, medicinal, and economic value in *C. oleifera*, are released from ripened fruits via capsule cracking, the volume of which increased fast from May to August and then nearly stopped after August [14]. The correct timing and degree of fruit dehiscence are essential for the harvesting and processing of *C. oleifera* seed oil, and for maximizing its value. Early fruit dehiscence leads to insufficient accumulation of valuable oil in seeds [15,16], whereas delayed dehiscing time increases the risk that the ideal fatty acid composition of seed oil will be changed [17]. Furthermore, low synchrony in fruit dehiscence of *C. oleifera* results in an additional procedure in actual production, the manual pick-up of seeds falling on the ground, which would certainly increase the production costs. In particular, with the approaching aging population, the rising labor costs are calling for a long-term challenge to the harvest of *C. oleifera*. Thus, cultivating and applying varieties with excellent cracking characters that are suitable for mechanization is a potential solution for optimizing the *C. oleifera* industry.

Extensive studies in *C. oleifera* have long been focused on germplasm collection and conservation, genetic breeding, cultivation, stress resistance, and synthesis of oil and other bioactive substances [18,19]. However, few studies have focused on fruit dehiscence in *C. oleifera*. To date, only several studies have focused on the evaluation of dehiscence traits in different varieties [20] and the identification of dehiscence-related homologous genes in *C. oleifera* [21,22]. Compared with model species, we barely know about the molecular control of dehiscence in *C. oleifera* due to the research deficiency in this filed. Here, a time-course transcription analysis of the developing fruits of the *C. oleifera* cultivar “CL40” was performed to understand the molecular basis of fruit dehiscence during ripening in *C. oleifera*. This study closed an important gap in our understanding of the transcription program and regulators controlling fruit dehiscence in *C. oleifera*.

## 2. Results

### 2.1. Overview of the Transcriptomic Analysis of Dissected Fruit Tissues

**Figure 1:**
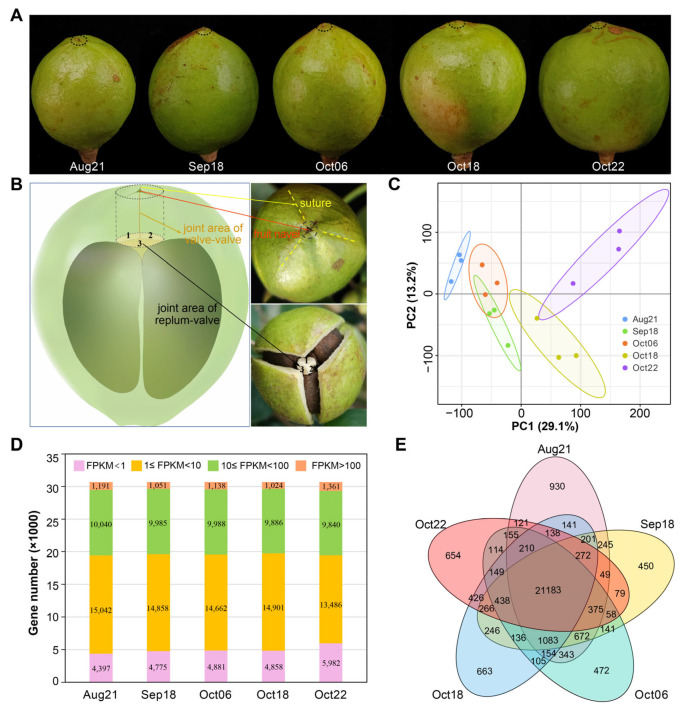
Plant tissue collection and global analysis of the pericarp–replum transcriptomes. (**A**) Observation of the *C. oleifera* fruit development at five different sampling dates. Dashed circles showed where the sampled tissues were incised. (**B**) Sample collection for RNA-seq. The cylindrical zone in the schematic corresponds to the tissue collected for high-throughput sequencing. (**C**) PCA plot of the RNA-seq data showed a clear separation among the samples. (**D**) Number of genes with different FPKM ranges at each stage. (**E**) Venn diagram showing the number of genes commonly and uniquely expressed among the five stages.

In *C. oleifera* fruits, dehiscence generally initiates in the apical part of the capsule (i.e., fruit navel, marked by the red arrow in Figure 1B), longitudinally along the sutures (Figure 1B, yellow dotted lines) during ripening. DZs and separation planes take place between the valves and the replum (Figure 1B, labeled by the number in black font), and between adjacent valves (Figure 1B, showed by the brown line). To explore the regulatory mechanism of fruit dehiscence during ripening in *C. oleifera*, pericarp–replum tissues that contained both the JAVV and JARV (Figure 1B, cylindrical zone shown in the schematic) at different developmental stages (August~October, Figure 1A) were collected and transcriptionally analyzed to determine the transcription dynamics.

A total of 30,669 genes were detected to express with FPKM values greater than 1.0 in at least one stage (Supplementary Table S2). All our subsequent analyses are based on this data matrix with these genes. PCA results show a transcriptional difference between different developmental stages (Figure 1C). For the sample of each stage, at least 76% of the genes were in the 1.0 to 100 FPKM range (Figure 1D). We used Venn diagram to show specifically or commonly expressed genes among different stages. In total, 21,183 genes (over 80% of the total 30,669 genes that expressed with FPKM ≥ 1 in each stage) were found to be shared in samples of all five stages (Figure 1E). These genes were functionally related to RNA binding, ribosome, spliceosome, and transcriptional activities (Supplementary Figure S1). We further examined the classification of 10,437 genes that were annotated as transcription factors (TFs) based on Pfam motifs. Pkinase, Pkinase_Tyr, RRM_1, p450, and NB-ARC were the top five abundant domain/motif categories (Supplementary Figure S2A, Supplementary Table S3). Given the crucial function in plant reproductive development, especially in floral organ differentiation, the MADS-box TF family was also noted (Supplementary Table S3, highlighted in yellow). Phylogenetic analysis identified the homologs of MADS-box members of *A. thaliana* (Supplementary Figure S2B).

### 2.2. Analysis of DEGs Suggested Drastic Transcriptional Changes Occurred in the Final Stages of Pericarp–Replum Tissue

**Figure 2:**
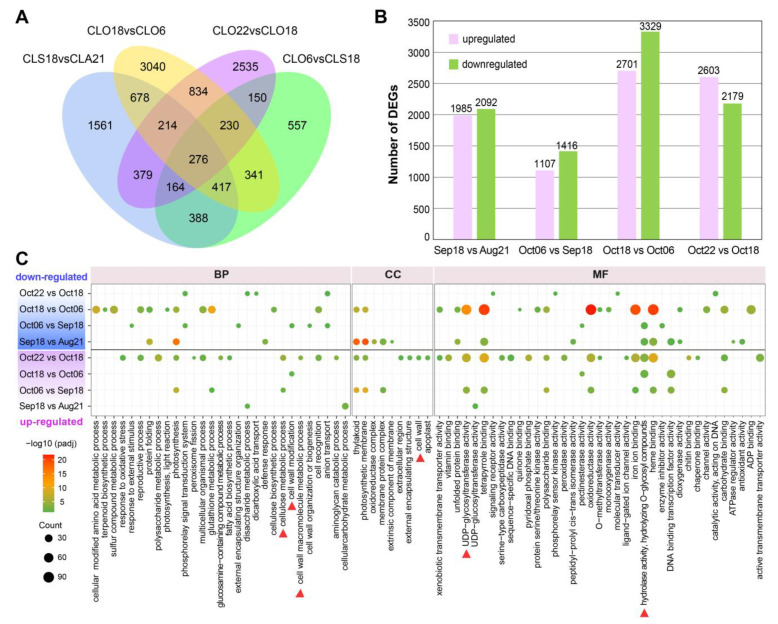
DEGs analysis of adjacent development stages in pericarp–replum tissue of *C. oleifera*. (**A**) Venn diagram showing the DEGs numbers in pairwise comparisons. (**B**) Histogram displaying number of DEGs up- and down-regulated in pair comparisons of adjacent development stages. (**C**) GO enrichment analysis of DEGs that are up-regulated and down-regulated in pairwise comparisons of adjacent development stages.

DEGs were identified from paired comparison of adjacent developmental stages by a fold change ≥ 2 and a *p*-value < 0.05, and 10,612 DEGs were ultimately obtained. Notably, more DEGs were exhibited in the two pairwise comparisons among the last three stages (Oct18 vs. Oct06, Oct22 vs. Oct18) (Figure 2A,B), suggesting drastic transcriptional changes in pericarp–replum tissues during the late stages of maturity. To explore the molecular differentiation during fruit development, these DEGs were further functionally classified through GO and KEGG pathway enrichment analysis. Compared to Oct18 and Oct06, where the down-regulated DEGs enriched the highest number of GO terms, more GO terms were found to be overrepresented in the up-regulated DEGs in the Oct22 vs. Oct18 pairwise comparison (Figure 2C). Strikingly, up-regulated DEGs of the pairwise comparisons, specifically Oct22 vs. Oct18 and/or Oct18 vs. Oct06, were distinctly enriched for GO terms of interest, such as cellulose metabolism, cell wall, UDP-glycosyltransferase, and hydrolase activity (Figure 2C, marked by red triangle), suggesting activity of cell wall components may function as potential factors affecting *C. oleifera* fruit dehiscence. These functional categories, linking to activity of cell wall, were significantly enriched for genes up-regulated at the last several stages, and illuminated us to focus more on transcriptional changes at the last two stages.

### 2.3. Time-Course Analysis of Pericarp–Replum Tissues Showed Strongly Induced Cell Wall-Related Transcriptional Changes in the Last Two Stages

**Figure 3:**
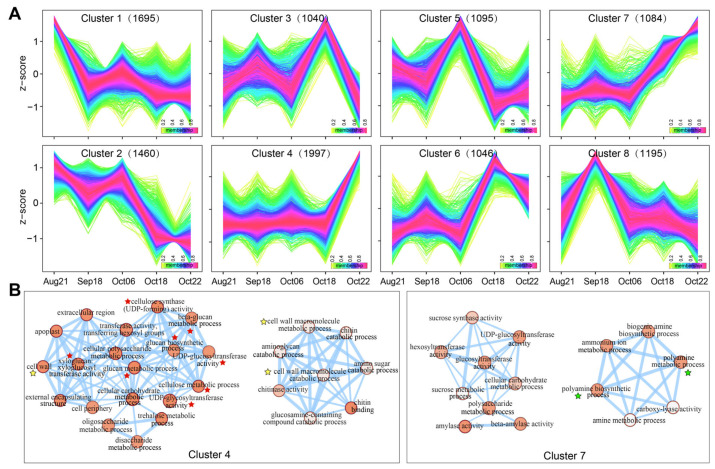
Clustering analysis and gene functional classification of sub-classes. (**A**) FCM clustering analysis of DEGs. The number in black font represents the number of genes assigned in each cluster. (**B**) GO enrichment analysis of the DEGs in Clusters 4 and 7. The weight of the connecting lines (edges) between nodes represents the number of genes overlapped between GO terms.

To determine the overall temporal characteristics of gene expression, we performed a fuzzy c-means algorithm (FCM) clustering analysis to group genes based on the similarity of their transcriptome profiles. A total of 11,764 DEGs (|log_2_FC| ≥ 1, *p*-value < 0.05 between adjacent stages) with an FPKM ≥ 1 in at least one stage among all five time points were clustered into eight sub-classes (cluster 1~8) (Figure 3A). Cluster 1 consisted of genes that were most enriched in functions related to photosynthesis processes, and were strongly down-regulated at the Sep18 stage (Supplementary Figure S3, and Supplementary Tables S4 and S5). We focused more on the DEG sets, which were more strongly expressed at the stages in October, especially the last two stages, that is, clusters 3, 6, 4, and 7 (Figure 3A). DEGs in Clusters 3 and 6 exhibited a roughly similar pattern in the first four periods with the strongest expression at the fourth stage, and then decreased. Clusters 4 and 7 consisted of genes that were strongly up-regulated at the last one and two stages, respectively. DEGs in Clusters 4 and 7 could be candidates for affording the strongest regulators of fruit dehiscence in *C. oleifera*. Functional enrichment analyses showed that multiple cell wall-related GO terms were significantly enriched for DEGs in Cluster 4, including cell wall and metabolic/catabolic processing of cell wall macromolecules such as cellulose, glucan, xyloglucan, and chitin (Figure 3B, marked by red and yellow stars). We also noted the enriched polyamine-related GO terms in Cluster 7 (Figure 3B, marked by green stars), suggesting a potential role of these growth regulators in fruit development, given their essential functions in plant reproductive development such as flowering, fruit growth, and senescence.

### 2.4. Co-Expression Network Analysis Revealed Relationships between Developmental Stages and DEGs

**Figure 4:**
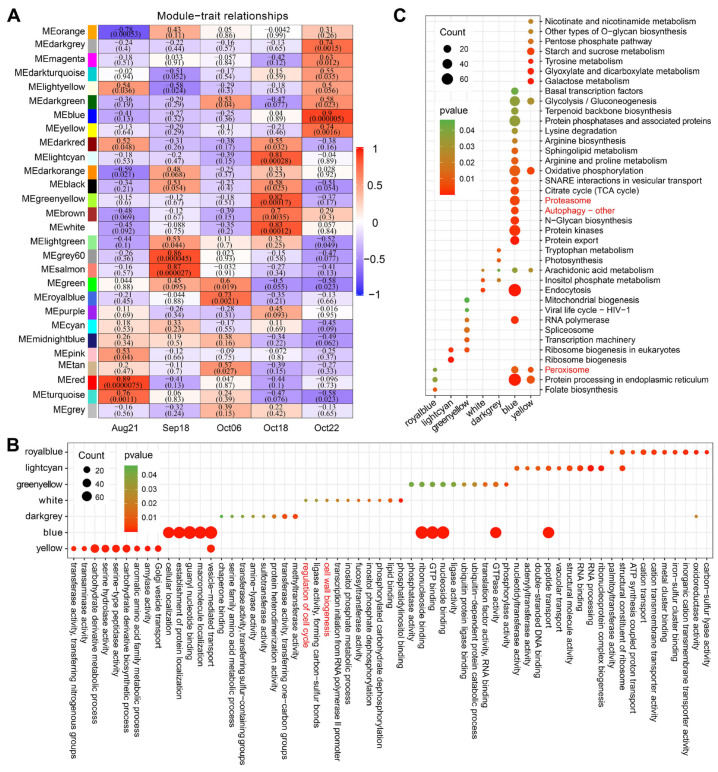
Detection of co-expression network in pericarp–replum tissues of *C. oleifera* fruit. (**A**) Module–tissue association matrix. The red or blue color of each cell in the matrix, respectively, indicates a positive or negative correlation between the module and the tissue. The values in cells indicate the correlation coefficient and the corresponding *p*-value (in parentheses). (**B**) Top 10 enriched GO classifications of genes in modules highly associated with the three developmental stages of October. Only modules that were significantly correlated with corresponding tissues at the 0.01 level, with a correlation coefficient greater than 0.7 were analyzed. (**C**) KEGG pathway enrichment analysis for genes of the same modules in (**B**).

To explore co-expression networks of genes, and relationships between the developmental changes and genes, we performed a WGCNA. The previously mentioned data set containing 30,669 genes was loaded into the WGCNA shiny App in TBtools, followed by data filtering twice with default parameters, and ultimately 19,361 genes were retained for subsequent analysis. The genes expressed in five sequentially developmental stages were ultimately classified into 28 distinct co-expression modules (Figure 4A and Supplementary Figure S4). Special focus was given to the modules that were identified as highly correlate with the last two developmental stages (Oct18 and Oct22) in October, including three Oct18-related modules (lightcyan, greenyellow, white), and three Oct22-related modules (darkgrey, blue, yellow), for which r^2^ was >0.7 and *p* was <0.01 (Figure 4A). The correlation between the module membership and the gene significance for corresponding module and stage are shown in Supplementary Figure S5.

Enrichment analyses were then performed to explore the biological functions of genes in these modules. The results showed that genes co-expressed in the white module were significantly enriched in cell wall and life cycle GO terms (Figure 4B, highlighted in red font). Proteasome and autophagy were the two main protein degradation pathways during plant senescence [23]. In total, 5019 genes in the blue module, corresponding to the last stage, were significantly enriched in KEGG pathways of proteasome, autophagy, and peroxisome (Figure 4C, highlighted in red font), reflecting that protein degradation systems were active late in fruit development to maintain proteostasis and export or salvage nutrients from senescing organs or tissues to develop reproductive parts.

### 2.5. Analysis of Cell Wall-Related Hub Genes in Co-Expressed Modules That Were Highly Associated with the Final Developmental Stage of C. oleifera Fruit Dehiscence

To explore the potential key stage-specific regulators, we identified the hub genes with the most connections in the networks of modules highly associated with two stages (Oct18 and Oct22), based on the standard settings kME > 0.90 and GS > 0.80. Multiple genes of interest, annotated as involved in cell wall activities, were identified as the hub genes of the co-networks of the yellow and blue modules (Supplementary Table S6, marked by yellow color).

**Figure 5:**
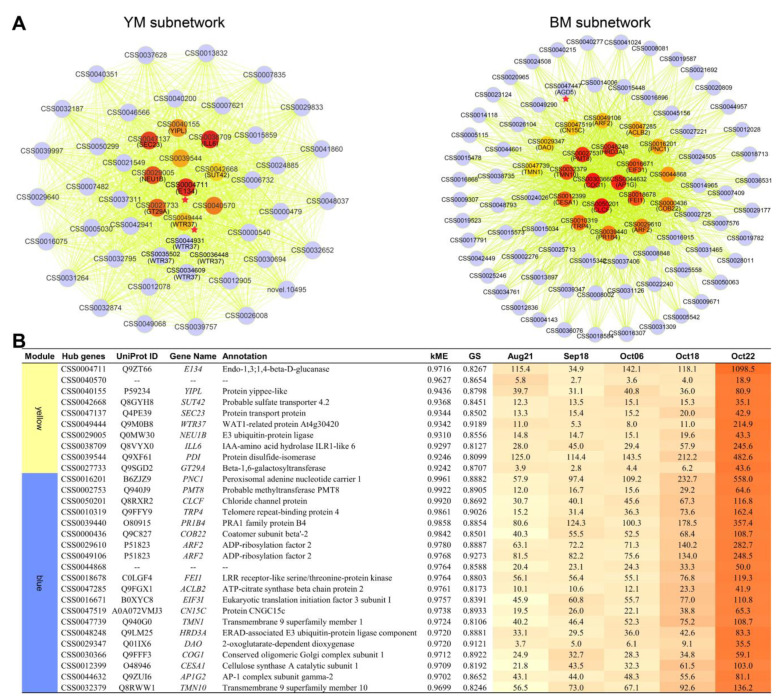
Analysis of hub genes in WGCNA modules. (**A**) Co-expression subnetworks of the hub genes in yellow and blue modules, highly related to the last stage of pericarp–replum tissue in *C. oleifera* fruit. The most highly connected 50 and 100 hub genes are shown. Nodes labeled with brown–red gradient indicate, respectively, the 10 and 20 top-ranked hub genes selected from the YM and BM subnetworks using Cytohubba, a plugin in Cytoscape. The calculated score of the corresponding nodes are shown with a color scheme from highly essential (red) to essential (brown). (**B**) Details of 10 and 20 top-ranked hub genes of the YM and BM subnetworks in (**A**).

To explore putative candidates with significant contributions, we then selected the most highly connected 50 and 100 hub genes from Oct22-associated yellow and blue modules, respectively, to generate the co-expressed subnetworks. To facilitate elaboration, the two newly generated subnetworks were named as YM and BM, respectively (Figure 5A). In YM and/or BM subnetworks, we noted several factors involved in metabolic and signaling pathways of plant hormones including auxin, cytokinin, brassinosteroid, and abscisic acid (Supplementary Table S7). Additionally, the YM subnetwork included multiple cell wall-associated factors, which included 1 Endo-1,3;1,4-beta-D-glucanase encoding gene (CSS0004711) and five that were annotated as WTR37 (Figure 5A). Similarly, in the BM subnetwork, genes related to cell wall formation and modification, i.e., synthesis of pectic compounds (CSS0019523), cellulosic compounds (CSS0012399), or involved in the signaling pathway that regulates cell wall function (CSS0018678, CSS0024026), were also noted in BM. In particular, we found the gene CSS0047447 (Figure 5A, red star-labeled node in BM subnetwork), annotated as coding for ADP-ribosylation factor GTPase-activating protein, had been reported to involve in cell separation during floral organ shedding and abscission zone cells.

To screen for more crucial core factors in the co-expression networks, we further used the MCC (Maximal Clique Centrality) topological algorithm of the Cytohubba plugin [24] in Cytoscape 3.9.1 to, respectively, obtain the 10 and 20 top-ranked hub genes of the YM and BM subnetworks (Figure 5A, nodes colored with a brown–red gradient). Details of the 30 top hub genes are shown in Figure 5B. We noted the top 10 hub genes of the YM subnetwork contain *E134* and 1 *WTR37* (Figure 5A, node labeled with a red star in YM subnetwork) as quoted in the preceding description, both of which were reported to mediate cell wall regulation. Among the top 20 hub genes of BM subnetwork, CSS0018678 and CSS0012399 encode LRR receptor-like serine/threonine-protein kinase FEI1 and Cellulose synthase A catalytic subunit 1, respectively. They also involved in the regulation of cell wall function. Specific changes in the cell wall component were an important aspect of pathways regulating fruit dehiscence. These cell wall-related genes were specifically expressed with their highest levels at Oct22, the end stage of fruit dehiscence during ripening, implying their potential role in *C. oleifera* fruit dehiscence.

### 2.6. Comprehensive Search for Potential Regulatory Factors Related to Fruit Dehiscence in C. oleifera

The mechanisms regulating fruit dehiscence during ripening have been intensively studied in annual crops of Brassicaceae and Fabaceae, for instance, *A. thaliana*, *B. napus*, and legume species. To better and more comprehensively understand of the transcription program and potential regulators of *C. oleifera* fruit dehiscence, we further retrieved our data, based on knowledge of dehiscent theme-related publications.

Two genes, CSS0030447 and CSS0027575, respectively annotated as *RPL* and *NST1* (*NAC SECONDARY WALL THICKENING PROMOTING FACTOR 1*), were down-regulated at the last two stages of *C. oleifera* pericarp development (Supplementary Figure S6). In addition, we identified two differentially expressed putative MADS-box genes, *SHP1* (CSS0030447) and *FUL* (novel.3794) (Supplementary Figure S2B), with the highest expression at stage Aug21 and Oct18, respectively (Supplementary Figure S6).

**Figure 6:**
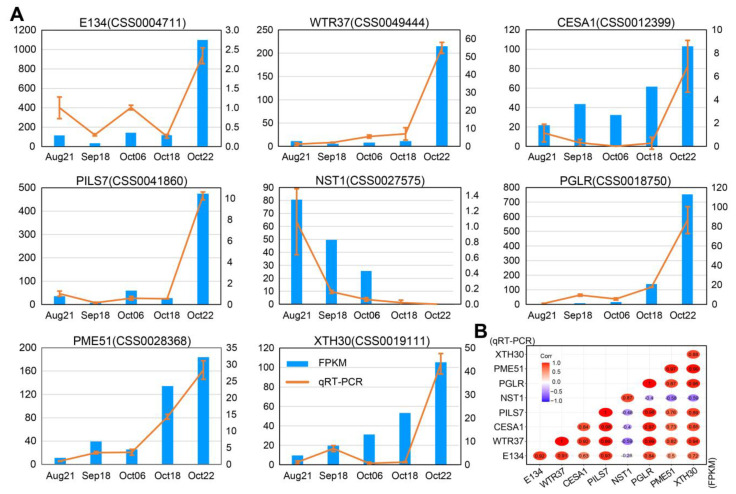
Validation of the DEGs expression by qRT-PCR. (**A**) Transcript abundance of DEGs quantified using qRT-PCR. For each gene, the relative expression level of each stage was compared to the first stage (Aug21). Genes expression levels detected by RNA-seq and qRT-PCR are shown in the left y-axis and right y-axis, respectively. Error bars indicate SD (*n* = 3). (**B**) Heatmap showing the correlation between RNA-seq and qRT-PCR outputs for the tested genes.

Consistent with how numerous of hydrolases were enriched in the DZ by the final stage of fruit dehiscence in *A. thaliana* [2], we found several putative hydrolases including polygalacturonase (PG)-, pectinesterase (PE)-, and xyloglucan endotransglucosylase (XET)-encoding genes, all of which reached their maximum expression level at the last stage (Oct22) (Supplementary Figure S6). The four PG-coding genes (CSS0014837, CSS0018750, CSS0024349, and CSS0002317) and five PE-coding genes (CSS0007314, CSS0028368, CSS0040974, CSS0044662, and CSS0015072) were also noted to be assigned to the yellow and blue module that were highly associated with Oct22 stage in WGCNA, respectively (Supplementary Figure S6). qRT-PCR confirmed the expression pattern of *PGLR* (CSS0030447) (Figure 6). PG has been shown to be essential for *Arabidopsis* fruit dehiscence [12]. The PE/pectin methylesterases and XET were also good candidates for fruit dehiscence regulation. These hydrolase genes that mediate cell wall modification were specifically overexpressed in the Oct22 sample, suggesting their potential roles in regulating fruit dehiscence during ripening of *C. oleifera*.

### 2.7. Quantitative Real-Time PCR (qRT-PCR) Assays of DEGs

To validate the reliability of RNA-seq data, we performed qRT-PCR analysis on the relative transcript abundance of eight DEGs, including one lignification-associated factor (*NST1*), and seven related cell wall-modifying genes (*E134*, *WTR37*, *CESA1*, *PGLR*, *PME51*, and *XTH30*) (Figure 5 and Supplementary Figure S6). The results showed that most of the eight genes tested were quantified to have expression profiles similar to those in RNA-seq data (Figure 6A). A high correlation was confirmed between the qRT-PCR and corresponding RNA-seq data, with Pearson’s correlation coefficients ranging from 0.84 to 1.00 (Figure 6B).

## 3. Discussion

### 3.1. Tissues Located in the Fruit Navel Region Are Likely to Be the Most Critical Structural and Functional Basis for the Dehiscence of C. oleifera Fruit

Extensive studies on species of Fabaceae and Brassicaceae, which are of major scientific and economic importance, provided our main knowledge of genetic control of fruit dehiscence. The fruit structure of the model plant *Arabidopsis* is typical of numerous species of Brassicaceae. The siliques of *Arabidopsis* consisted of with two longitudinally fused valves, which joined to the replum, and a putative seed-bearing septum [4]. By contrast, the pod of legumes has a single folded carpel lacking a replum [4], and it is usually open on the dorsal side of the pod at maturity more frequently than on the ventral side [25,26,27]. Although greater force is needed to break the ventral side in mature pods, there are only the fiber cap cells to connect the valve edges on the dorsal side [26]. That is, fruit dehiscence tends to start at the zone of weakness. The fruit dehiscence of *C. oleifera* initiates typically at the fruit navel (Figure 1A, dashed circle), and continues along the suture (Figure 1B, yellow broken lines). The pericarp at the apex of the fruit appears to be the weakest point of the *C. oleifera*. Here, the replum is attached to the valves at multiple junction areas (Figure 1B, indicated by numbers), binding the latter to avoid separation. We reasoned that tissues located in the fruit navel region serve as the most critical structural and functional basis for the dehiscence of *C. oleifera* fruit. Therefore, in this study, tissues of the navel region, containing both the replication membrane valve junction area and the fruit umbilical valve junction area (the cylindrical zone in the schematic in Figure 1B), were collected for our research.

### 3.2. Dehiscing Control in the Later Stage of C. oleifera Fruit Development Points to Cell Wall Activity

In current known genetic models controlling fruit dehiscence, several master patterning genes direct the correct differentiation and establishment of the DZ in the valve margin to ensure the structural basis required for dehiscence [28]. On the other hand, downstream of these genes, several cell wall-modifying genes act on the lignification layer and separation layer, promoting dehiscence through establishing mechanical tension and weakening cell adhesion [5,28]. 

Given that our research object has effective functional structures driving a normal dehiscence, much attention attached to TFs that control the DZ differentiation in early fruits is not necessary. In fact, a relatively small number of the upstream master patterning genes were detected from the data, sequenced from tissues sampled from fruits during the enlargement stage to those almost matured. In contrast, transcriptional evidence of GO enrichment and co-networks directed the late development of *C. oleifera* pericarp towards cell wall separation (Figure 2C, Figure 3B and Figure 5). Functional classification links to cell wall were significantly enriched (labeled by a red triangle in Figure 2C, and red or yellow stars in Figure 3B) by DEGs that drastically changed during the last sampling interval (Figure 2A,B and Figure 3A). Additionally, co-network analysis indicated genes (E134, WAT37, FEI1, and CESA1) encoding enzymes/proteins associated with cell wall modification were identified as the core members of co-expressed modules positively correlated with the end stage (Oct22) of *C. oleifera* fruit dehiscence (Figure 5A,B; Supplementary Table S7). Endo-1,3;1,4-beta-D-glucanase specifically degrades cell wall (1,3) (1,4)-beta-D-glucans [29]. *WAT1* (*WALLS ARE THIN 1*) encodes a plant-specific, novel auxin transporter and regulates the secondary cell wall formation via auxin signaling pathway [30]. *wat1* mutants revealed little to no secondary cell walls in fibers [30]. In pods, pod dehiscence for seed dispersal requires the secondary wall deposition in extraxylary fibers [31]. *FEI1* is involved in regulating cell wall function, such as cellulose biosynthesis [32]. *CESA1* encodes one of the subunits of cellulose synthase required for the crystallization of beta-1,4-glucan microfibril, a major mechanism of the cell wall formation.

These results suggested activities related to the cell wall were the most evident biological process in the dehiscence progression in *C. oleifera* fruit during ripening. This is consistent with changes in cell wall modification and the activities of associated enzymes observed in the ripening–dehiscence of *Arabidopsis* [33] and *Akebia trifoliata* [34] fruits, as well as physical cracking of *Litchi chinensis* [35] and tomato [36] fruits. However, in dehiscing fruit of *C. oleifera*, we did not notice remarkable transcriptional events reflecting tissue lignification, another essential cytological process required for fruit dehiscence in Brassicaceae and legumes.

### 3.3. Hydrolases Targeting the Cell Wall Macromolecular Components Are Important Factors in the Dehiscence of C. oleifera Fruit

Although the fruits of plant species are high diverse in morphology, texture, and maturity, the cytological processes of lignification and separation in the DZ that determine fruit dehiscence over the maturation are common. In our data, analysis of functional enrichment and co-networks reflected that the most strikingly biological event during the fruit dehiscence of *C. oleifera* was related to cell wall activity. It is known that hydrolase-mediated weakening of intercellular adhesion and the degradation of cell walls, resulting in cell loosening prior to cell separation, is an essential process during dehiscence in fruits of many plants of Brassicaceae and legumes [2,37].

Cellulose, hemicellulose, and pectin are the three main types of components of plant cell walls [38]. These enzymes are known to be involved in abscission of fruit, seeds, and flower organs, as well as other cell separation processes such as fruit and anther dehiscence [2,13,27]. The cellulase gene *CEL6* and the hemicellulase gene *MAN7* have been confirmed to promote silique dehiscence in *A. thaliana* [13]. Another study also demonstrated that hemicellulose gene *BnMAN7A07*, a homolog of *Arabidopsis MAN7* from rapeseed, manipulated the dehiscence-resistance in *B. napus* [39]. Pectinases were thought to be of functional conservation in cell separation during abscission and dehiscence, and were expected to play a major role in [13]. The pectinase genes *ADPG1* and *ADPG2* encode PG were previously reported acting on the separation layer by weakening cell adhesion and were essential for *A. thaliana* fruit dehiscence [5]. Our results showed that genes, annotated as coding for PG and PE, were identified to be highly positively related to the late stage (Oct22) of fruit development of *C. oleifera* (Figure 4A and Supplementary Figure S6). PE is another candidate that is determinant of fruit dehiscence. PG mediates the demethylation of homogalacturonan, one of the structural domains of pectin, making pectin easier to be degraded by pectin-degrading enzymes [40]. XTH is another enzymatic activity related to wall loosening widely involved in fruit ripening and softening [41,42]. Despite the lack of direct genetic evidence for XTH involvement in fruit cracking, in the end stage of fruit development in both *B. napus* and *C. oleifera* (Supplementary Figure S6), the XTH coding gene was also specifically up-regulated in the DZ [43,44]. Hydrolases mediating the cell wall macromolecular components are important factors, which are likely to be targeted by exogenous compounds for controlling dehiscence of *C. oleifera* fruit artificially.

## 4. Methods

### 4.1. Sample Collection and Histological Analysis

A 7 mm diameter sharpened, thin-walled cylindrical steel cutter was applied to excavate tissues vertically at the navel of the fruit of the “CL40” *C. oleifera* cultivar (Figure 1A). The corer penetrated deeply up to the seed shell, ensuring both the joint area of valve-valve (JAVV) and the joint area of replum-valve (JARV) were contained in collected samples (Figure 1B). Each of the three biological replicates per stage was taken from an independent fruit. In 2021, samples were taken weekly during August, September, and at 3 day intervals in October. Each of the three biological replicates per stage was taken from an independent fruit and frozen in liquid nitrogen and stored at −80 °C.

### 4.2. High-Throughput Transcriptome Sequencing

Pericarp–replum tissues with both JAVV and JARV collected on 21 August, 18 September, 6 October, 18 October, and 22 October (named refer to corresponding sampling date, i.e., Aug21, Sep18, Oct06, Oct18, and Oct22) were selected for total RNA extraction. Total RNA was isolated from pericarp using the RNAprep Pure Plant Kit (TIANGEN, Beijing, China), and was concentration-measured and quality-assessed using the Agilent Fragment Analyzer 2100 system. RNA-Seq library building and subsequent Illumina Hiseq-PE150 sequencing were performed at NOVOGENE China. 

### 4.3. Sequence Assembly, Functional Annotation, and Classification

Sequence assembly was based on the reference genome of “Shuchazao” (*Camellia sinensis* var. *sinensis*) [45] using HISAT2 2.0.5. New transcripts were assembled with StringTie (1.3.3b) and were functionally predicted based on the Pfam, SUPERFAMILY, GO, and KEGG databases. The read count for each gene was obtained using featureCounts (v1.5.0-p3) and the FPKM values were used to calculate and analyze the gene expression abundances. Differential expression analysis was carried out using DEseq2 v1.20.0. The functional enrichment analysis and principal component analysis (PCA) was performed using Novomagic Cloud Platform (https://magic.novogene.com/customer/main#/homeNew, (accessed on 24 July 2023)).

### 4.4. Gene Clustering and Weighted Gene Co-Expression Network Analysis (WGCNA)

The analysis of gene expression pattern was performed using the TCseq R package, with a random seed set as 123 [46]. The co-expression network was conducted using the WGCNA Shiny plugin in the TBtools toolkit [47]. Soft-thresholding power was set as 8, and other parameters were followed with the default settings. Cytoscape 3.9.0 was employed to visualize networks.

### 4.5. Quantitative RT-PCR (qPCR) Assays

The qRT-PCR was performed using the same samples as RNA sequencing. Reverse transcription was performed using the PrimeScript™ 1st Strand cDNA Synthesis Kit (TaKaRa, Dalian, China) according to the manufacturers’ instructions. Data were collected from three biological replicates and analyzed with the 2^−ΔΔCT^ method [48]. A “GAPDH-q” gene in *C. oleifera* [49] was selected as the reference gene. The primer pairs are presented in Supplementary Table S1. The qRT-PCR programs were referenced from a previous study [50] and performed on the ABI PRISM 7300 real-time PCR system (Applied Biosystems, Foster City, CA, USA) with TB Green Premix Ex Taq II (Tli RNaseH Plus) (TaKaRa, Dalian, China).

## 5. Conclusions

In this study, we revealed the transcriptional program and potential regulators controlling fruit dehiscence over maturation in *C. oleifera*. Our analysis provided insights into the cell wall macromolecule-mediated the fruit dehiscence during ripening in *C. oleifera*. The roles of these putative cell wall modification genes in *C. oleifera* fruit dehiscence should be targeted by reverse genetics in future studies.

## Data Availability

Raw reads in this study have been submitted to NCBI under bioproject (PRJNA1014923).

## Supplementary Materials

The following supporting information can be downloaded at https://www.mdpi.com/article/10.3390/plants12183314/s1. Figure S1: Functional enrichment analyses of the shared genes in all five stages. Figure S2: Analysis of transcription factors (TFs). Figure S3: KEGG pathway enrichment analysis of DEGs in corresponding clusters identified by fuzzy c-means algorithm (FCM) clustering analysis. Figure S4: Hierarchical clustering dendrogram shows co-expression modules identified by WGCNA. Figure S5: Scatter plot reflect the correlation between the module membership and the gene significance for corresponding module and stage in WGCNA. Figure S6: Expression profiles of the putative homologous genes of *C. oleifera* were shown in the model for silique dehiscence regulating in A. thaliana. Table S1: Primer sequences for quantitative real-time PCR (qRT-PCR). Table S2: Gene expression analysis by RNA-Seq. Table S3: Statistics and classification of the putative transcription factors (TFs) in each sample. Table S4: Table view of GO functional categories significantly enriched of DEGs in corresponding clusters identified by fuzzy c-means algorithm (FCM) clustering analysis. Table S5: Table view of KEGG pathways significantly enriched of DEGs inin corresponding clusters identified by fuzzy c-means algorithm (FCM) clustering analysis. Table S6: Details of the hub genes in each module that were highly associated with corresponding stages.
