# Supplementary material for: Transcriptomic Time-Course Sequencing: Insights into the Cell Wall Macromolecule-Mediated Fruit Dehiscence during Ripening in Camellia oleifera
Source: Plants (Basel). 2023 Sep 20;12(18):3314. doi: 10.3390/plants12183314 (PMC10535178; doi:10.3390/plants12183314)
Supplement: Supplementary file 1 [file plants-12-03314-s001.zip › Supplementary Figures S1-S6.pdf]

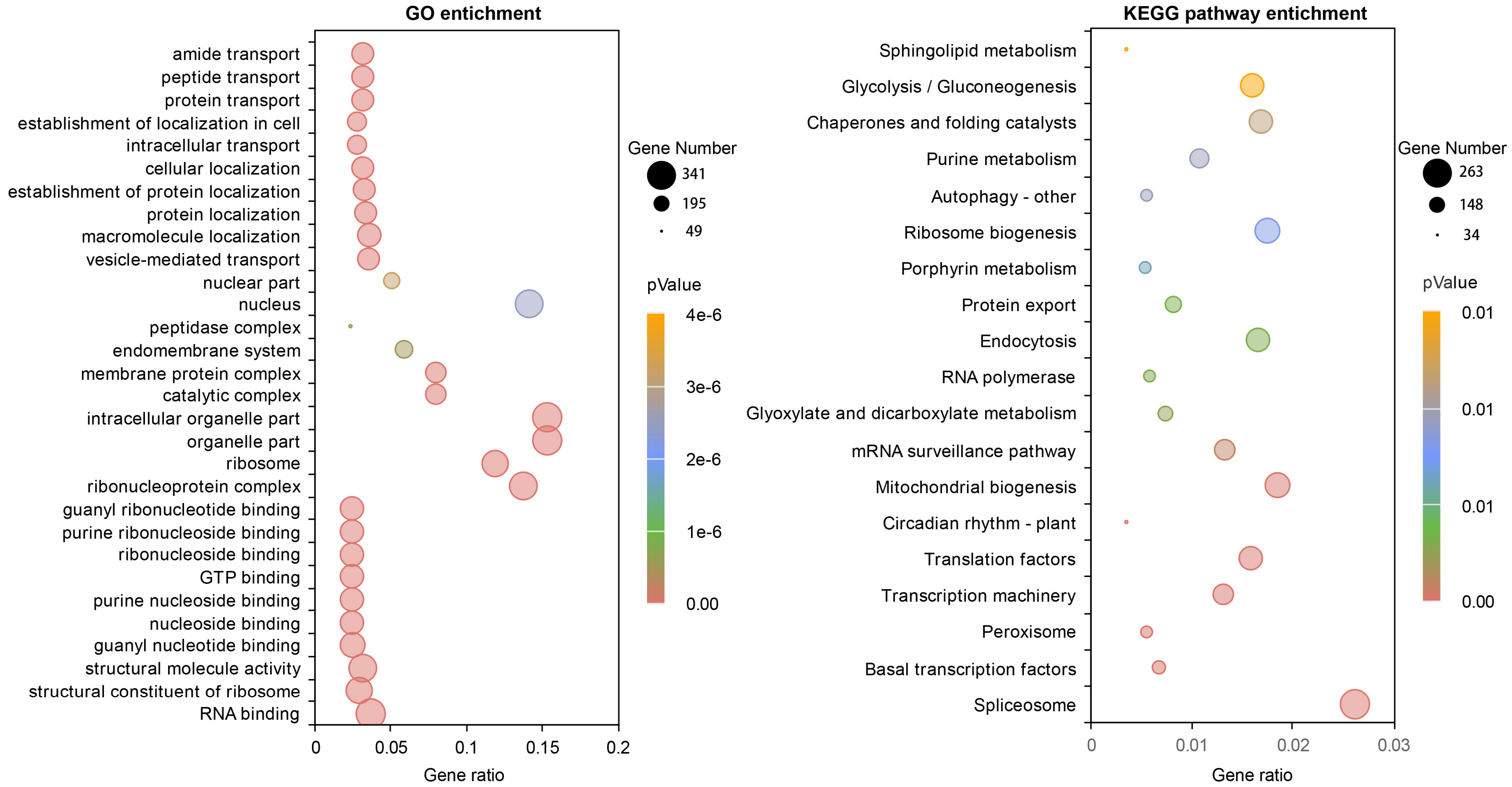

Supplementary Fig. S1 Functional enrichment analyses of the shared genes in all five stages. TOP 30 and 20 function categories of the GO and KEGG pathway enrichment analysis were shown, respectively.

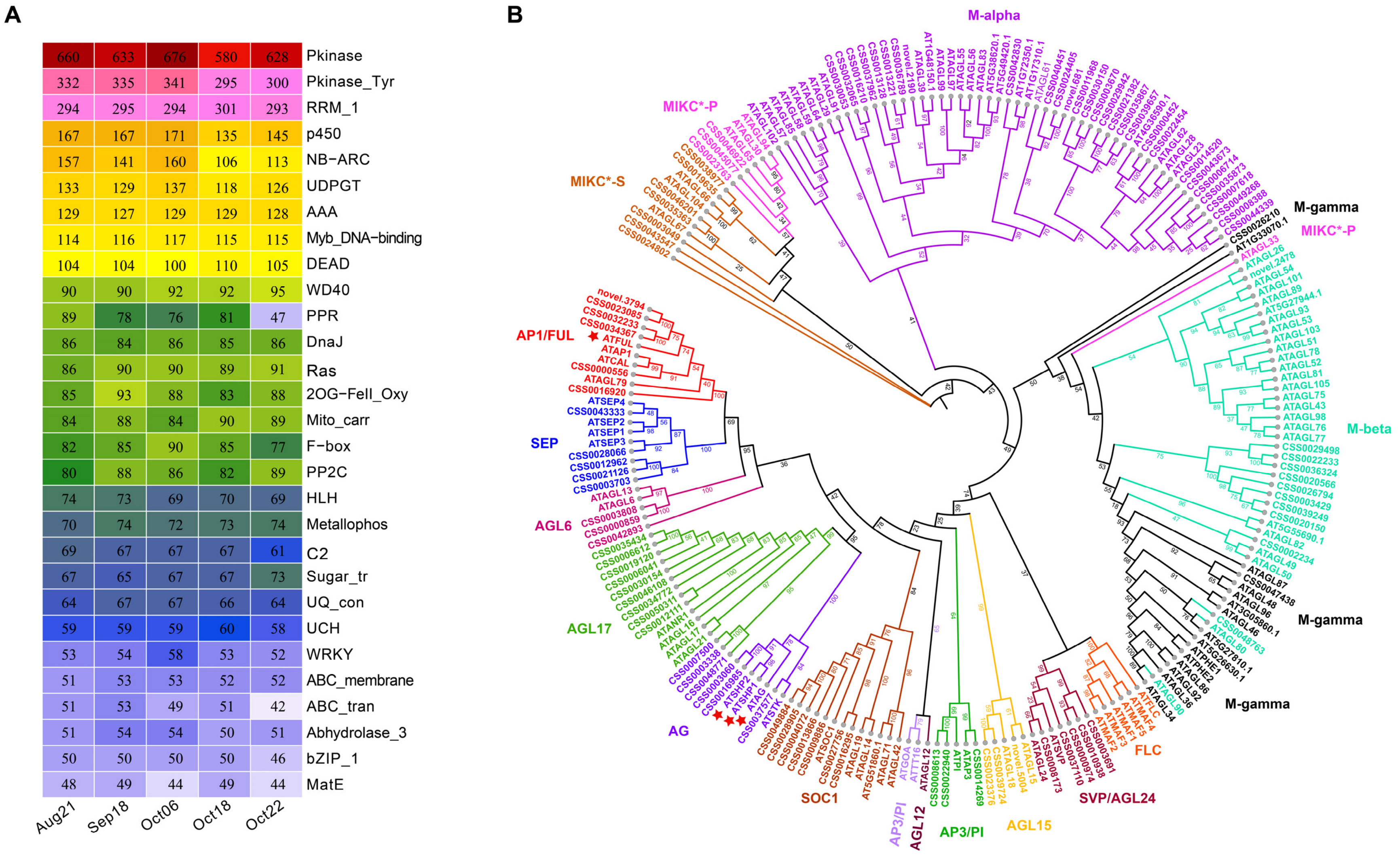

Supplementary Fig. S2 Analysis of transcription factors (TFs). (**A**) Heatmap shows the domain categorization of genes annotated as transcription factors with an expression level of FPKM ≥ 1 in each stage. Only the first 30 rows in Supplementary Table S2 were shown. (**B**) Phylogenetic analysis of MADS-box genes in *A. thaliana* and genes that were annotated as MADS-box transcription factors in *C. oleifera*. The Phylogenetic tree was constructed based on the translated amino acid sequences of 99 genes annotated as MADS-box TFs in *C. oleifera* and the protein sequence of 108 *A. thaliana* mads-box genes, using the Phylogenetics function in TBtools software with default parameters.

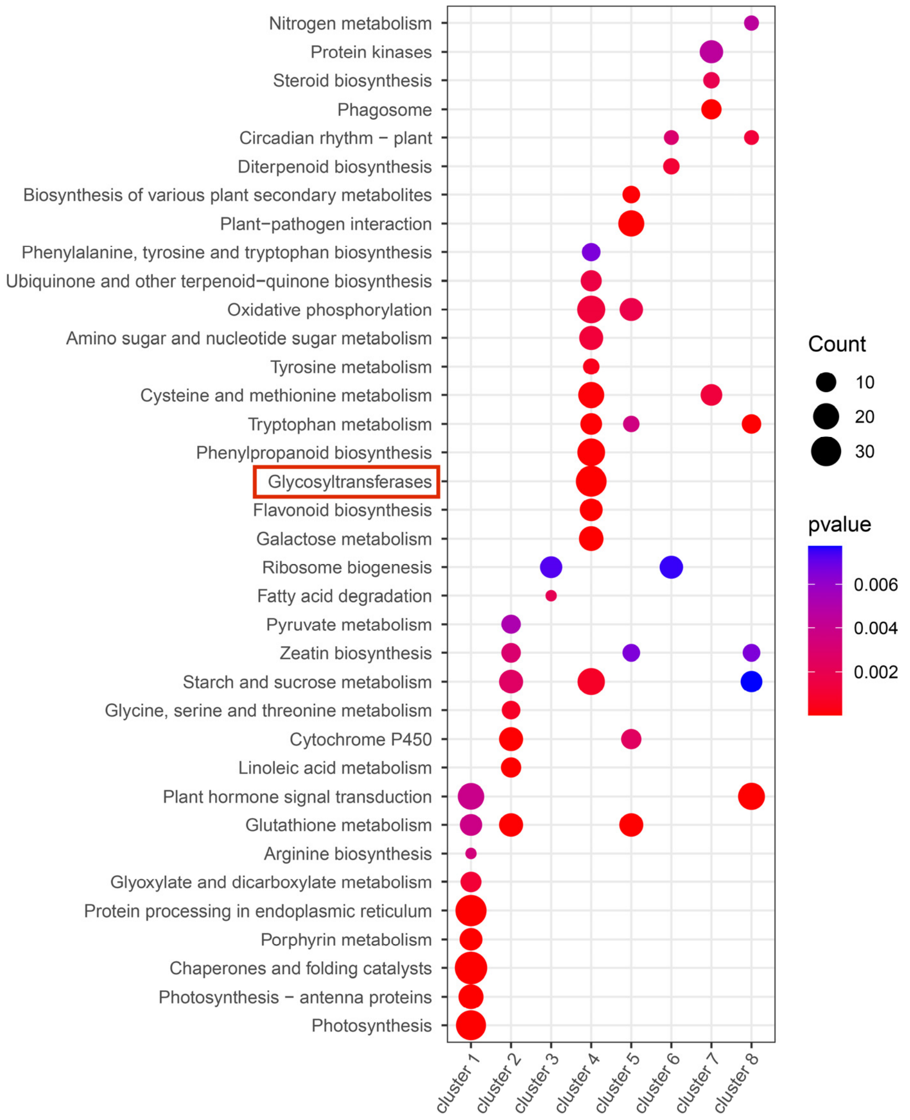

Supplementary Fig. S3 KEGG pathway enrichment analysis of DEGs in corresponding clusters identified by fuzzy c-means algorithm (FCM) clustering analysis. Pathways with p-value < 0.01 were showed.

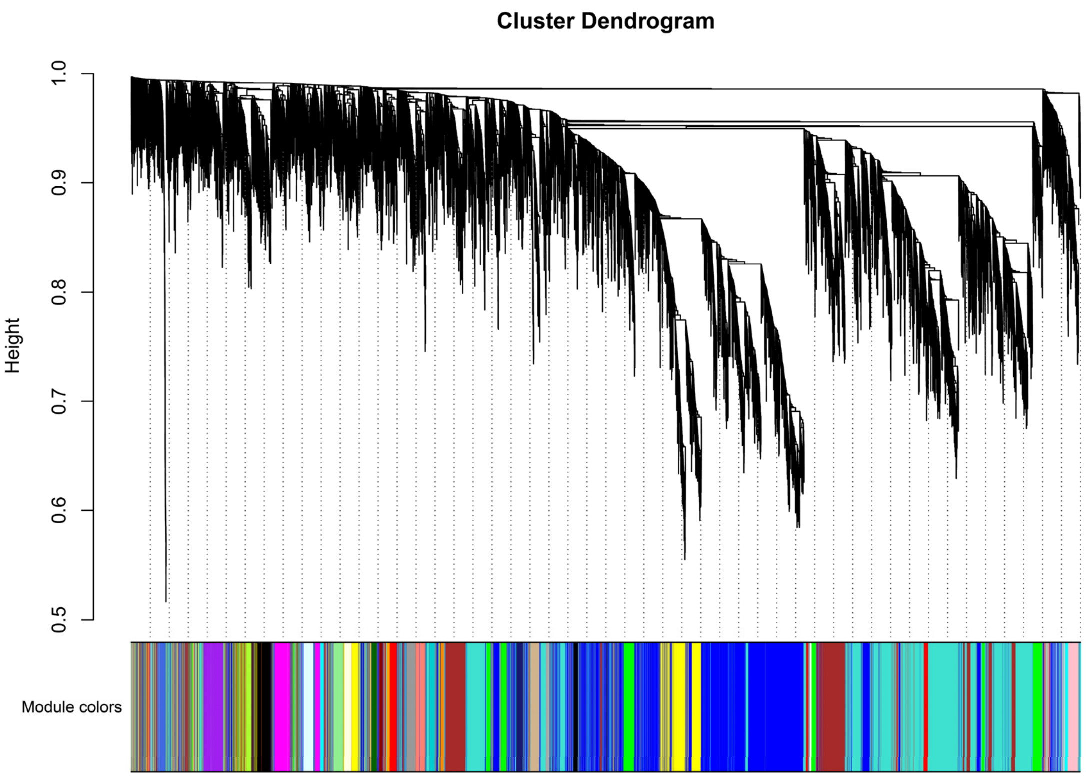

Supplementary Fig. S4 Hierarchical clustering dendrogram shows co-expression modules identified by WGCNA. Each branch (black vertical line) in the dendrogram represents an individual gene. The major tree branches constitute 28 distinct co-expression modules, labeled with different colors underneath the dendrogram. Each color of the colored bars under the dendrogram corresponds to a module with highly similar expression patterns of genes.

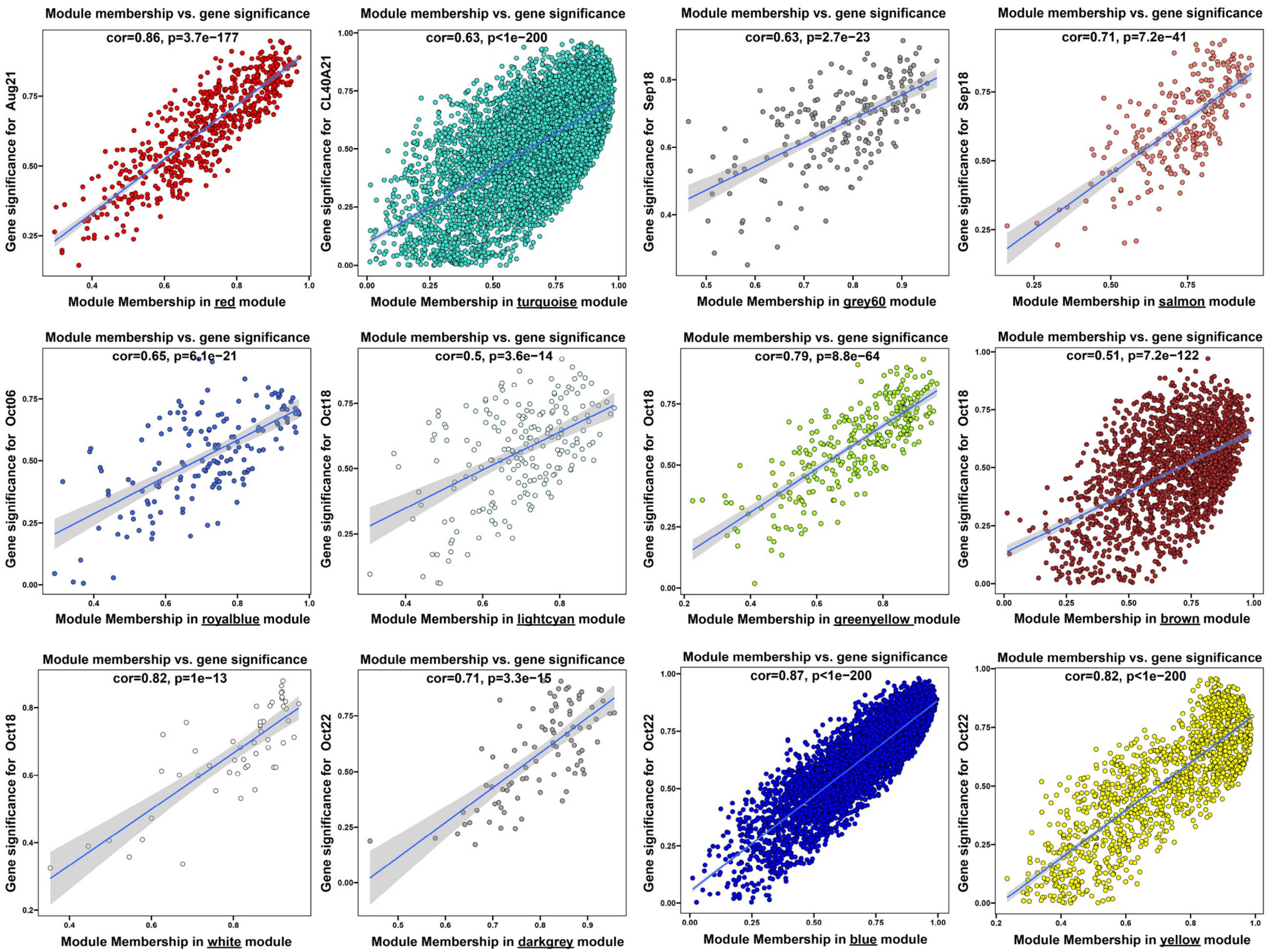

Supplementary Fig. S5 Scatter plot reflect the correlation between the module membership and the gene significance for corresponding module and stage in WGCNA. Module membership and gene significance represent the correlation between genes and modules, as well as between genes and stages, respectively.

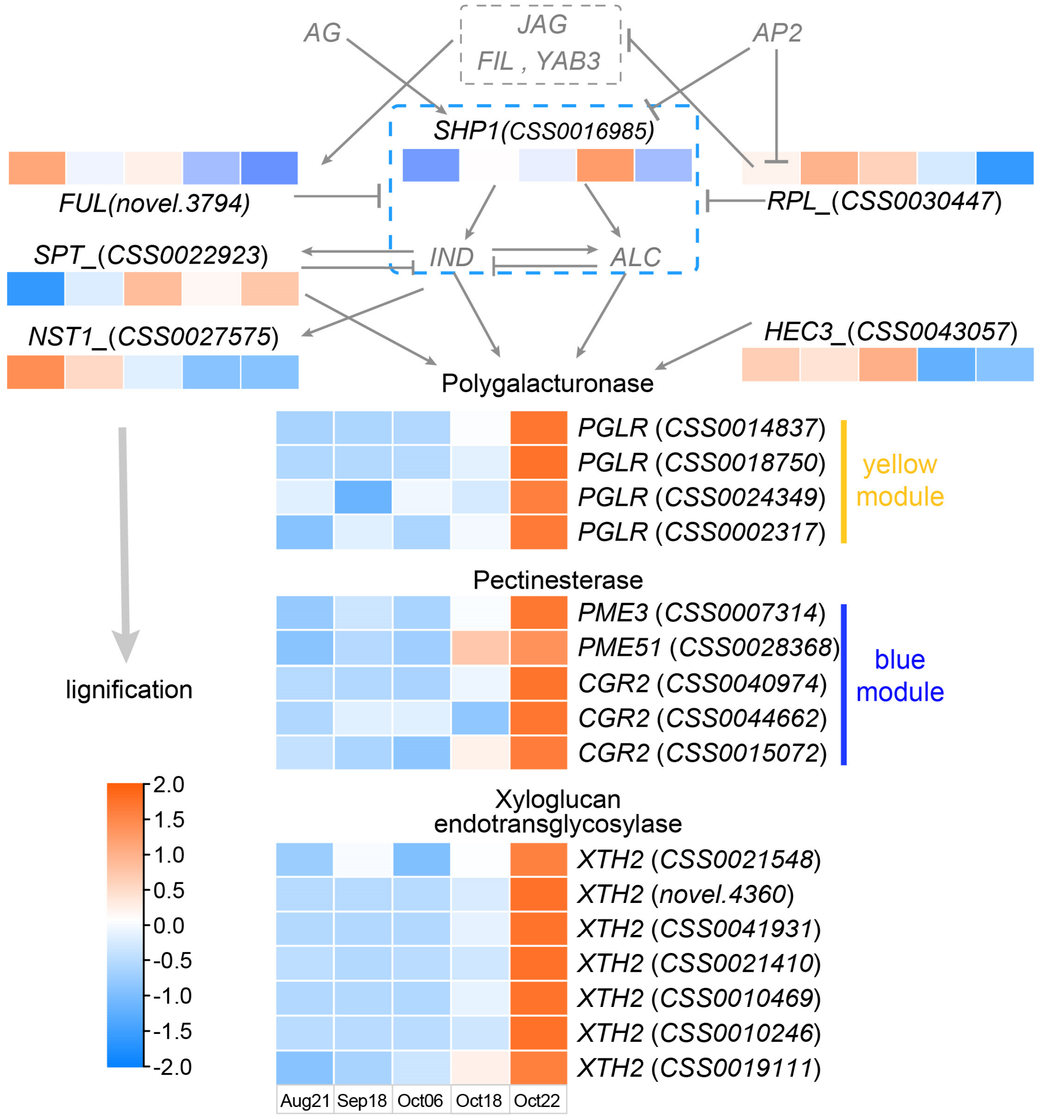

Supplementary Fig. S6 Expression profiles of the putative homologous genes of *C. oleifera* were shown in the model for silique dehiscence regulating in *A. thaliana*. The heat map showed the relative expression levels of the searched genes. Genes in gray font are not differentially expressed or not known in *C. oleifera*. Arrows indicate positive regulation and bars indicate process inhibition.
